# Analysis Strategy for Identifying the *O*-Linked Glycan Profile and *O*-glycosylation Sites on Recombinant Human Follicle Stimulating Hormone-*C*-terminal Peptide (rhFSH-CTP)

**DOI:** 10.3390/molecules30102141

**Published:** 2025-05-13

**Authors:** Xinyue Hu, Yuxing Xiang, Xiaoming Zhang, Yue Sun, Yi Li, Lvyin Wang, Ping Lv, Zhen Long, Chenggang Liang, Jing Li

**Affiliations:** 1 National Institutes for Food and Drug Control, Beijing 102629, China; huxinyue@nifdc.org.cn (X.H.); zhangxiaoming@nifdc.org.cn (X.Z.); sunyue@nifdc.org.cn (Y.S.); liyi2016@nifdc.org (Y.L.); wanglvyin@nifdc.org.cn (L.W.); lvping@nifdc.org.cn (P.L.); chenggangliang@nifdc.org.cn (C.L.); 2 School of Life Science and Technology, China Pharmaceutical University, Nanjing 210009, China; q344320257@163.com; 3 ThermoFisher Scientific Corporation, Beijing 100080, China; longzhen8866@126.com

**Keywords:** FSH-CTP, *O*-glycan, *O*-glycosylation sites, core 1, HCD, ETD

## Abstract

*O*-glycosylation is a common post-translational modification on extracellular and secreted proteins driving biochemical and biophysical interactions at the cell surface. Glycosylation affects drug immunogenicity, efficacy, and clearance, making it a critical attribute of biotherapeutics. Unlike *N*-linked glycans, *O*-linked glycans are difficult to characterize because there is no consensus sequence for glycosylation sites on the polypeptide and a universal enzyme to release *O*-glycans from proteins. To overcome these hurdles, *O*-glycan analysis and localization require an appropriate and well-validated approach, particularly for recombinant human follicle stimulating hormone-*C*-terminal peptide (rhFSH-CTP). FSH-CTP consists of a native FSH α/β subunit fused with the *C*-terminal fragment of a human chorionic gonadotropin (hCG) β subunit, which is heavily *O*-glycosylated. However, few FSH-CTP *O*-glycosylation identification methods exist. Thus, we developed a characterization method for the *O*-linked glycan profile and glycosylation sites of rhFSH-CTP. By means of *O*-glycan profiling, we identified predominantly core 1-based structures with good reproducibility. For site-specific localization, the *O*-glycopeptidase OpeRATOR, used with sialidase, helped identify *O*-glycosylated peptides. Electron transfer/higher-energy collision dissociation (EThcD), combined with OpeRATOR, identified all six glycosylation sites. This approach improves quality control for rhFSH-CTP biosimilars and other CTP-fusion proteins, contributing to the development of standardized *O*-glycan identification methods.

## 1. Introduction

As one of the most common post-translational protein modifications, glycosylation affects various protein functions, such as cell signaling, adhesion, communication, and molecular trafficking [1,2]. Glycosylation, i.e., the conjugation of carbohydrates with a protein backbone, impacts the structure and function of certain proteins. Glycosylation presents heterogeneity in two critical dimensions: macroheterogeneity (site-specific glycosylation) and microheterogeneity (structural diversity of glycans at occupied sites), which both profoundly influence protein stability, pharmacokinetics, and biological activity. Characterizing and quantifying glycosylation is important for disease diagnosis and biotherapeutic manufacturing [3,4]. Additionally, glycosylation is a critical quality attribute of biotherapeutics due to its role in drug immunogenicity, efficacy, and clearance. Indeed, even small changes in the glycosylation sites and/or glycan types may dramatically affect the efficacy and safety of a glycosylated drug product. However, the complexity of glycan composition and structure remains a challenge for glycosylation analysis.

*N*-linked and *O*-linked glycans are the most common protein-bound glycans [5]. While *N*-glycan analysis techniques are becoming routine procedures, *O*-glycan analysis remains challenging. Compared to the *N*-glycosylation pathway, which mediates the attachment of *N*-acetylglucosamine (GlcNAc) to some asparagine side chain residues at Asn-Xaa-Ser/Thr sequons (Xaa represents any amino acid except proline) [6], *O*-glycosylation shows significantly greater variability in glycosylation sites and glycan structures. Many glycoproteins carry glycans initiated by GalNAc attached to the hydroxyl of Ser or Thr residues. Mucins are the class of glycoproteins carrying the greatest number of *O*-GalNAc glycans. But unlike *N*-glycosylation, mucin-type *O*-glycosylation lacks a universal consensus motif. This is attributed to the diversity of polypeptide GalNAc-transferases (GalNAc-Ts, over 20 isoforms in humans), each exhibiting distinct substrate preferences for specific sequence contexts or structural motifs. These enzymes collectively contribute to the heterogeneous initiation of *O*-glycosylation, with subsequent core structures (e.g., cores 1–4) formed through stepwise elongation by additional glycosyltransferases, further diversifying glycan complexity. These core structures serve as the foundation for building more complex glycans, capable of forming mature linear or branched structures. Common constituents of *O*-GalNAc glycans include GalNAc, Gal, GlcNAc, Fuc, and Sia, while Man, Glc, and Xyl are not typical components [7]. Therefore, the absence of a unified consensus sequence, coupled with the combinatorial action of multiple enzymes, complicates the prediction and identification of *O*-glycosylation sites, making their identification more challenging. Furthermore, while various enzymatic tools exist for the cleavage of *N*-glycans, such as PNGase F [8], the release of *O*-glycans is limited to short oligosaccharide versions of core 1/core 3 mucin-type *O*-glycans [9,10]. The lack of a universal enzyme that can release intact complex *O*-glycans further complicates the characterization of *O*-glycosylation [11]. To overcome these technical hurdles, developing an appropriate approach for *O*-glycan and *O*-glycosite analyses is essential.

Mass spectrometry (MS) characterization could provide important information about *O*-glycan structure and *O*-glycosite location within a protein [12,13,14]. In the context of *O*-glycan structure analysis, several options for the chemical release of intact *O*-glycans have been reported, such as β-elimination and hydrazinolysis [15,16]. The reducing end of glycans can then be coupled with labeling reagents (2-aminobenzoic acid (2-AA) and 2-aminobenzamide (2-AB), which contain fluorophores) detectable by hydrophilic interaction chromatography with fluorescence detection and MS (HILIC-FLD-MS) [17,18,19]. These chemical release methods present long completion times (at least 16 h) and unavoidably produce peeling products [20]. Kameyama et al. [21] have shown that the *O*-glycan yields of hydroxylamine combined with the superbase 1,8-diazabicyclo[5.4.0]undec-7-ene were comparable to those of hydrazinolysis techniques, with minimal peeling (3%). They used the chemical cleavage method commercialized as the EZGlyco *O*-glycan Prep Kit to release *O*-glycans through eliminative oximation [22]. Locating *O*-glycosylation sites involves cleaving glycoproteins into shorter peptides/glycopeptides using proteolytic enzymes (e.g., trypsin, Glu-C, or multi-enzyme) [23,24]. The peptides are then identified using MS/MS with different electron-based fragmentation (such as electron transfer higher-energy collisional dissociation (HCD), electron transfer dissociation (ETD)/electron capture dissociation (ECD), and ETD with HCD supplemental activation (EThcD)) [25]. For example, MS methods, such as chemical labeling of endogenous glycan structures, lectin enrichment of glycans from native or glycoengineered cells, or *O*-GalNAc-specific endo-peptidase treatment, can directly identify *O*-GalNAc glycan modification sites in glycoproteins. Furthermore, the use of mucin-type *O*-proteases, like OpeRATOR and StcE, which specifically cleave the *N*-terminal end of an *O*-glycosylated Ser/Thr, represents a significant advance in glycosylation site mapping [26,27].

Human chorionic gonadotropin (hCG) is a glycoprotein hormone secreted by placental trophoblasts and trophoblastic tumors. It is found in the blood and urine of women during pregnancy. The CTP of hCG contains 28 amino acids (^118^SSSSKAPPPSLPSPSRLPGPSDTPILPQ^145^) and four *O*-glycosylation sites (S121, S127, S132, and S138) [28,29], which bear mucin-type GalNAc-core structures. These sites were identified by subjecting peptides to alkaline treatment to detect alkali-labile serine residues, then performing Edman degradation and subtractive analysis to localize glycosylated positions, and finally carrying out amino sugar composition analysis (e.g., galactosamine detection in βT13 and βT15). Even though it did not use modern tools, such as MS, this approach provided foundational evidence through systematic peptide mapping and chemical reactivity studies [30]. Advances in MS have enabled more precise and efficient methods for the identification of *O*-glycosylation sites in hCG. Using high-resolution liquid chromatography–mass spectrometry (LC–MS) employing multiple dissociation methods and Tandem Mass Tag (TMT 10plex) labeling, Zhu H et al. [31] identified *O*-glycosylation sites in hCG. Bai et al. [32] cleaved *N*-linked glycans from hCG using PNGase F and then treated it with a mixture of exoglycosidases to partially remove *O*-linked glycans while retaining GalNAc residues. They identified the *O*-glycosylation sites of hCG via LC–MS/MS by detecting a characteristic 203 Da mass increment associated with *O*-glycosylated peptides, confirming previously known sites. Biskup K et al. [33] mapped hCG *O*-glycosylation sites using in-gel ammonia-based glycan release, microcolumn purification, and permethylation, followed by MALDI-TOF MS and GlycoPeakfinder analysis. This approach identified core 1- and core 2-type *O*-glycan compositional differences between CGB3/5/8 and CGB7 variants.

**Figure 1:**
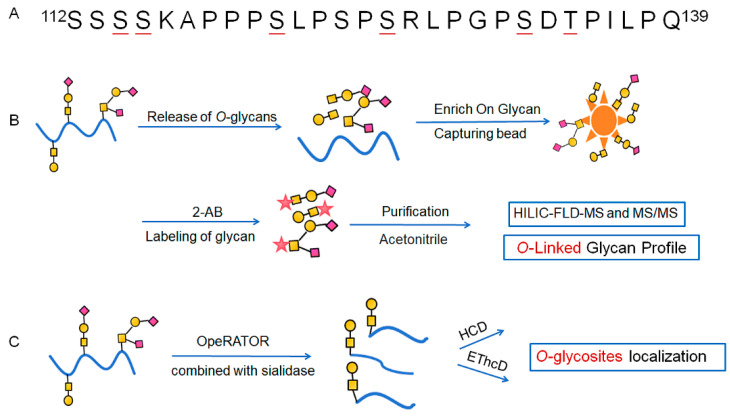
Workflow for *O*-linked glycan profiling and localization on FSH-CTP. (**A**) CTP amino acid sequence: ^112^SSSSK APPPS LPSPS RLPGP SDTPI LPQ^139^. Underlined residues are previously reported *O*-glycosylation sites. (**B**) Characterization of *O*-linked glycan profile by HILIC-FLD-MS after *O*-glycan release, labeling, and purification with the EZGlyco *O*-glycan Prep Kit. (**C**) Localization of *O*-glycosylation sites by HCD or EThcD.

The FSH-*C*-terminal peptide (FSH-CTP) can be produced in Chinese Hamster Ovary (CHO) cells through recombinant DNA technology. FSH-CTP has the same pharmacodynamic profile as FSH but with a markedly prolonged activity, achieved by adding the CTP of hCG to the β-subunit of human FSH. FSH-CTP (corifollitropin alfa [Elonva^®^], Schering-Plough) is currently the only CTP-containing product approved for clinical use, indicated in the structural diagram of the drug manual with 6 theoretical *O*-glycosylation sites. Among the potential modification sites (Figure 1A), two additional modification sites were identified within the sequence ^112^SSSSKAPPPSLPSPSRLPGPSDTPILPQ^139^, compared with the CTP of hCG. However, few methods for identifying the *O*-glycosylation of FSH-CTP or its biosimilar, especially through MS, have been reported. Furthermore, there is no standardized method for identifying *O*-glycans in domestic and foreign pharmacopeias, restricting the structural analysis of *O*-glycosylation of glycoprotein drugs and structure–function studies, hindering the go-to-market process of such drugs.

Based on high-resolution MS and current analytical techniques, we employed an *O*-glycosite localization and *O*-linked glycan profiling strategy for FSH-CTP. We released *O*-linked glycans from FSH-CTP and labeled them with 2-AB using the EZGlyco *O*-glycan Prep Kit and found that core 1-based structures were predominant within CTP *O*-glycans (Figure 1B). To localize *O*-glycosylation sites, we integrated the specificity of an OpeRATOR from *Akkermansia muciniphila*, combined with sialidase, to digest the CTP. This enzymatic treatment generated core 1-containing glycopeptides and retained the *O*-glycosylated serine or threonine residue at their *N*-terminus. We then employed methods involving both HCD and EThcD fragmentation to investigate *O*-glycosite localization (Figure 1C). This *O*-glycopeptide identification strategy offers a potentially effective approach for sample preparation of FSH-CTP and its biosimilars and aids detailed *O*-glycosylation characterization. These results may contribute to the development of standardized *O*-glycan identification methods for CTP-fusion proteins.

## 2. Results and Discussion

### 2.1. Determination of O-Glycan Profiles of FSH-CTP by HILIC-FLD-MS and MS/MS

**Figure 2:**
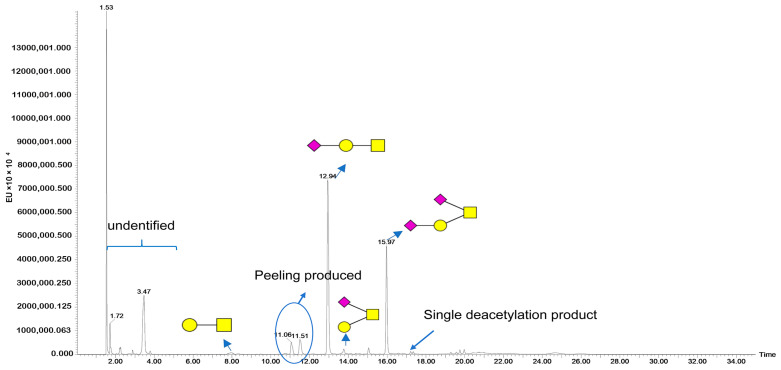
Fluorescence chromatogram of *O*-glycan types and peaks, obtained from protein samples (60 μg initial protein quantity) analyzed by HILIC-FLD-MS. The illustrated glycan structures omit the 2-AB fluorophore. The yellow square represents *N*-acetylgalactosamine (GalNAc). The yellow circle represents galactose (Gal). The red rhombus represents *N*-Acetylneuraminic Acid(NeuNAc).

**Figure 3:**
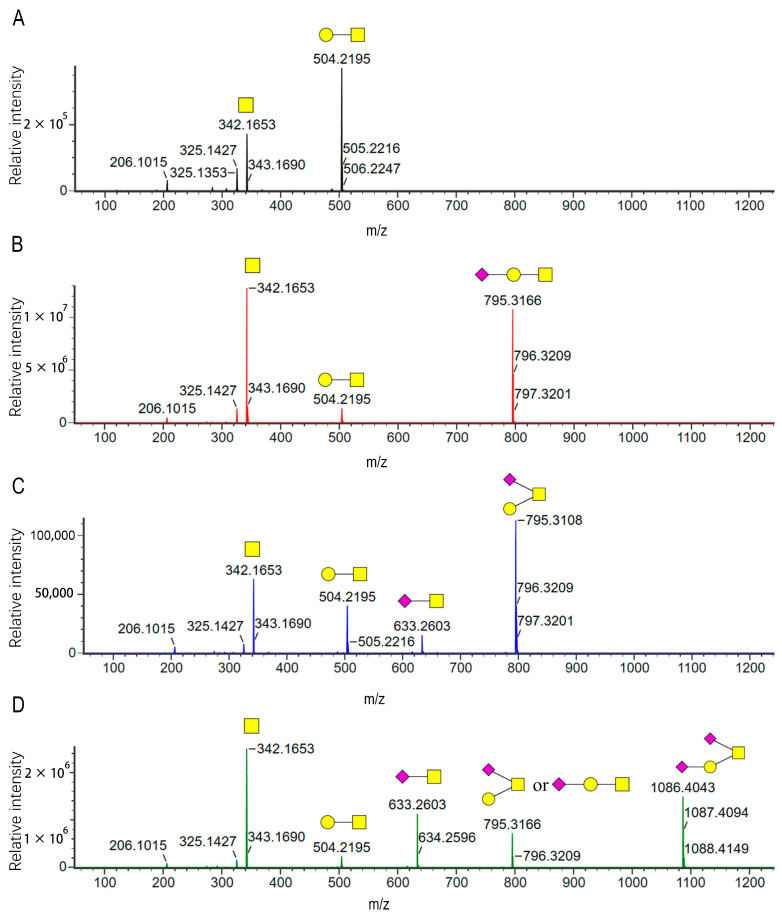
MS/MS spectra of the four major glycoforms. (**A**) GalGalNAc. (**B**) NeuAcGalGalNAc. (**C**) Gal(NeuAcGalNAc). (**D**) NeuAcGal(NeuAcGalNAc). The illustrated glycan structures omit the 2-AB fluorophore.

Figure 1B illustrates the experimental procedure. Glycoproteins reacted with DBU (1,8-diazabicyclo[5.4.0]undec-7-ene) and 50% hydroxylamine to release intact *O*-glycans. Next, the aldehyde of the reducing end of glycans is covalently bound to hydrazide on a capture enrichment column. The glycans were then subjected to 2-AB derivatization on the capture enrichment column and released from the beads of the column before the excess derivatization reagent was removed. Figure 2 shows the elution patterns of *O*-glycans from FSH-CTP by HILIC-FLD. We identified the structures of *O*-glycans from FSH-CTP by HILIC-FLD-MS and MS/MS. We analyzed mass spectra using UNIFI 2.0.194.0 software and validated the assignments through manual inspection. We identified four main *O*-glycans attached on the peptides (Table 1), with *m*/*z* values of 504.22, 795.32, 795.32, and 1086.41 (Figure S1). We tentatively identified the structures of these four glycoforms by matching their masses with the *O*-glycan library and adducts of 2-AB (*m*/*z* 139). However, the identity and linkages of the glycan units cannot be determined from the mass measurements alone [34], particularly given the potential for isomeric structures based on the MS data. Therefore, we performed MS/MS analysis and identified the predominant *O*-glycans in FSH-CTP as extensions of the core 1 structure. We observed linear and branched structures, including monosialylated core 1, branched monosialylated core 1, and disialylated core 1. Based on MS/MS data, these structures correspond to the observed *m*/*z* values, 504.22 (GalGalNAc, core1, Figure 3A), and 1086.41 (NeuAcGal(NeuAcGalNAc), disialylated core 1 by Figure 3D). The *m*/*z* 633.26 fragment ion (Figure 3C) allows the differentiation of the two *m*/*z* 795.32 isomers at MS1. These isomers are 795.32 (NeuAcGalGalNAc, monosialylated core 1, retention time 12.94 min, Figure 3B) and 795.32 (Gal(NeuAcGalNAc), isomer of monosialylated core 1, retention time 13.77 min, Figure 3C).

Other peaks with different retention times could also represent identical glycan structures, but these are not the predominant *O*-glycan types of CTP. Peeling is a process where the released non-reduced glycan undergoes degradation from the reducing core monosaccharide. The peaks at retention times of 11.06 min (NeuAcGal, *m*/*z* 592.23) and 11.51 min (NeuAc, *m*/*z* 430.18) are byproducts of the peeling process. This reaction typically occurs under alkaline conditions with a nucleophilic attack on the released glycan’s terminal sugar, initiating an elimination reaction involving the C-2 hydrogen of the open-ring form of the core, generating an alkene between C-2 and C-3, destabilizing the β1-3 glycosidic linkage, and separating the core from the proceeding monosaccharide. Additionally, there are also trace amounts of mono-deacetylated products. The peak with a retention time of 15.05 min (*m*/*z* 753.31) corresponds to the mono-deacetylated NeuAcGalGalNAc. The two peaks with retention times of 17.21 and 17.35 min (*m*/*z* 1044.40) are both mono-deacetylated products of NeuAcGal(NeuAcGalNAc).

Next, we calculated the peak area percentage for GalGalNAc, NeuAcGalGalNAc, Gal(NeuAcGalNAc), and NeuAcGal(NeuAcGalNAc) using normalized peak integration. The spectrum of the blank solution processed in parallel showed no significant interference peaks affecting glycan identification, demonstrating the good specificity of the method (Figure S2). Adjusting the flow rate to 0.1 mL/min still allowed us to separate and detect the four major glycan types (Figure S3).

**Figure 4:**
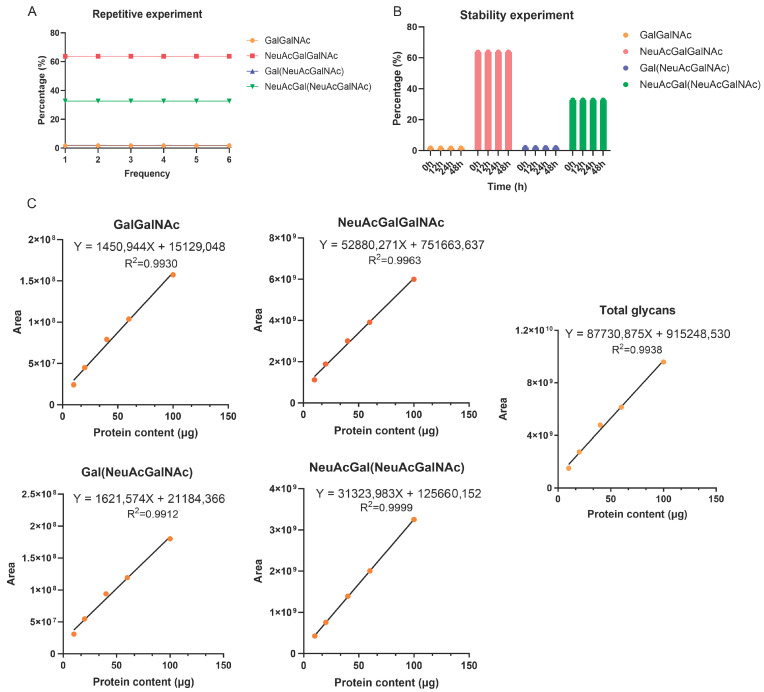
Methodological validation experiments for the characterization of *O*-oligosaccharide chains. (**A**) Reproducibility experiment with six replicate samples (60 μg of protein). (**B**) Stability experiment with samples (60 μg of protein) stored for up to 48 h. (**C**) The linear equation for all four glycan types and the total glycan type.

Subsequently, we assesse d the reproducibility and sample stability of our method. We prepared 60 µg protein samples in parallel to evaluate reproducibility. We calculated the relative standard deviation (RSD) of the peak area percentage for each glycan peak and the total peak and found that the RSD for the peak area percentages of the four glycan types (*n* = 6) was below 1.5% (Figure 4A). To assess the storage stability of the derivatized glycans, we dissolved the already derivatized glycans obtained from an initial 60 μg protein sample in acetonitrile and transferred them to LC vials. We then stored these vials at 4 °C and analyzed their contents after 0, 12, 24, and 48 h. The proportion of each *O*-glycan type remained stable over the tested time period, indicating that the derivatized glycans are stable under these storage conditions (Figure 4B).

A linear regression analysis with protein quantity as the X-axis and the peak areas of various glycan types as the Y-axis yielded a linear correlation coefficient (R^2^) greater than 0.99 for each of the four glycan types and all four glycan types together. This result indicates a very strong linear relationship, and LC–MS measurements showed high linearity from 10 to 100 μg of FSH-CTP (Figure 4C). Next, we wondered about the reliability of the recovery rate for evaluating the accuracy of analytical methods. To some extent, the enrichment, labeling, and cleaning processes of glycans could affect the recovery rate. The recovery rate is calculated as: recovery rate = (peak area measured at a certain protein amount)/(theoretical peak area at this protein amount) × 100%, with theoretical peak area at this protein amount = peak area measured at 40 μg sample × (protein amount/40). By analyzing five samples with different protein amounts and calculating the recovery rates for both the total peak area and the peak areas of the four glycan types, we found that the overall recovery rate ranged from 70% to 130% (Table 2), which sits within the generally acceptable range for bioanalytical method validation. The width of this recovery rate range reflects the inherent variability in glycan enrichment and derivatization efficiency, particularly given the complexity of biological matrices and sample preparation processes. The recovery rates were higher at lower initial protein amounts (10 and 20 µg) and lower at higher initial protein amounts (60 and 100 µg) (Table 2). Among the glycoforms analyzed at varying initial protein concentrations, we observed slight variations in the percentage of peak areas for Neu5AcGalGalNAc and Neu5Ac[Neu5AcGal]GalNAc (Table S1). Next, we performed peak integration and area normalization on the four glycoforms and their byproducts at different initial protein concentrations. We found that the percentage of peak areas for peeling and single deacetylation remained relatively stable for varying initial protein concentrations (Table S2), indicating that the degradation of major glycoforms was not the primary driver of the observed changes in recovery and proportion. This phenomenon can be attributed to several factors: at low concentrations, the excess of 2-AB reagent could cause an overestimation of labeling efficiency. Meanwhile, high concentrations may saturate the binding sites on the beads, preventing the capture of some target glycans. Even though the peak area of each component shows a linear relationship with concentration (R^2^ > 0.99), the response factors (i.e., peak area per unit concentration) of different components may vary substantially. When processing glycans from low protein amounts (10 or 20 µg), the peak areas of some components may approach the detection limit (low signal-to-noise ratio), increasing integration errors (e.g., small peaks being obscured by noise). Our initial method linearity experiments showed that GalGalNAc and Gal(NeuAcGalNAc) were not detected when the initial protein concentration was below 10 µg, indicating that they were below the detection limit.

*O*-linked glycan profiling revealed that the predominant *O*-glycoforms are core 1-derived structures. This observation allowed us to analyze *O*-glycosylation sites using glycopeptides modified with core 1 structures, potentially providing an effective approach for site-specific analysis. However, glycan profiling also showed some variability in the peak area percentages of individual glycoforms with different initial protein concentrations. While each glycoform exhibited acceptable linearity, this variability warrants consideration for relative quantitation. Furthermore, we did not fully evaluate the sensitivity of this method and its performance relative to alternative *O*-glycan release techniques for low-abundance glycoforms.

### 2.2. Determination of O-Glycosylation Sites Localization by HCD

**Figure 5:**
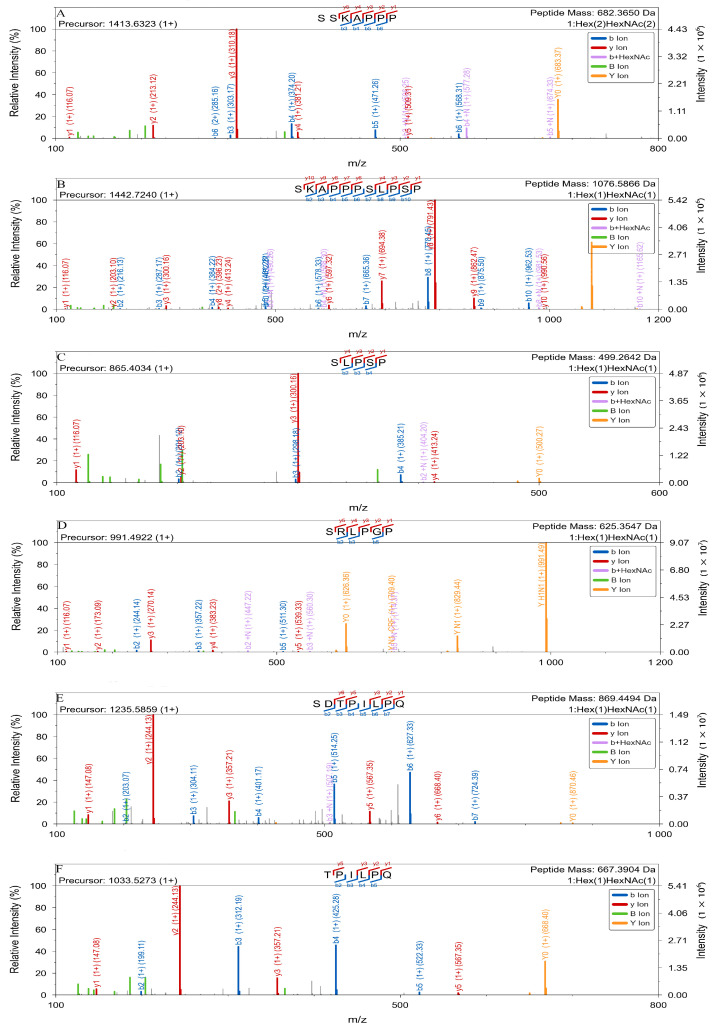
HCD fragmentation MS/MS of *O*-glycopeptide truncations using OpeRATOR and sialidase cotreatment. HCD fragmentation MS/MS spectra of the *O*-glycopeptides SSKAPPP (**A**), SKAPPPSLPSP (**B**), SLPSP (**C**), SRLPGP (**D**), SDTPILPQ (**E**), and TPILPQ (**F**).

HCD is a beam-type collision-induced dissociation that has been used for peptide characterization for decades. Alving K et al. [35] described a nanoelectrospray ionization (ESI)-quadrupole time-of-flight (QTOF)-MS/MS method for characterizing *O*-glycosylation sites in MUC2 glycopeptides. In a single experiment, this method allows to determine glycosylation sites and linked carbohydrate moieties and differentiate isobaric glycopeptide structures. Medzihradszky KF et al. [36] developed a new MS method for characterizing *O*-linked glycopeptides without chemical degradation and successfully determined carbohydrate structure and peptide sequence in some cases. Hanisch FG et al. [37] used QTOF ESI MS/MS to pinpoint *O*-glycosylation sites (Thr9 & Thr21) on a MUC1 glycopeptide, leveraging accurate y-/b-ion analysis with simpler spectra than PSD-MALDI MS. Müller S et al. [38] used QTOF ESI MS and Edman degradation to analyze MUC1 from T47D cells and found high-density *O*-glycosylation at all five potential sites and frequent amino acid sequence variations in the repeat domain. Chalkley RJ et al. [39] utilized QTOF MS to identify *O*-GlcNAcylation sites with femtomole sensitivity, enabling direct, derivatization-free glycosylation site identification. They also used QTOF MS/MS to identify novel *O*-GlcNAc sites on the serum response factor protein and found two new sites and a new phosphorylation site [40]. The *O*-glycopeptide from CTP, with the sequence ^112^SSSSKAPPPSLPSPSRLPGPSDTPILPQ^139^, contains nine potential *O*-linked glycosylation sites (S/T residues). The reported *O*-glycosylation sites within FSH-CTP include S114, S115, S121, S126, S132, and T134; they are all located on the CTP chain. Digesting the CTP with trypsin only yields two or three *O*-glycopeptides, and one *O*-glycopeptide can contain multiple *O*-glycosylation sites. For example, S114 and S115 are adjacent *O*-glycosylation sites, which further increases the difficulty of analysis. The OpeRATOR enzyme is a specific *O*-glycopeptidase that theoretically cleaves *O*-GalNAcylated proteins or peptides into glycopeptides with an *O*-glycan at the *N*-terminus. Its mechanism of action allows it to convert multiply *O*-glycosylated peptides into multiple singly *O*-glycosylated peptides, facilitating site-specific characterization. Additionally, the combined use of sialidase enzyme to remove sialic acids from *O*-glycans significantly enhances the cleavage efficiency of OpeRATOR, leading to optimal results. The OpeRATOR enzyme exhibits activity towards a sialylated core 1 and core 3 *O*-glycans, and removing sialic acids is crucial for achieving optimal enzymatic activity. Here, *O*-linked glycan profiling revealed that treating FSH-CTP with sialic acid retained core 1 structures; therefore, to determine the *O*-glycosylation sites of FSH-CTP, we combined HCD fragmentation with OpeRATOR enzymatic digestion and searched *O*-glycopeptide sites on glycopeptides containing core 1 glycans identified and characterized using the Pglyco 3.1 software. We first noted that two glycopeptides (SRLPGP and TPILPQ) possessed only a single potential *O*-glycosylation site each. The *m*/*z* of the *O*-glycopeptide SRLPGP was 991.4922, while the theoretical *m*/*z* of the unmodified peptide segment was 625.3547. Therefore, the modification was GalGalNAc (*m*/*z* 365). The MS/MS spectrum showed the y ions (y5) at *m*/*z* 539.33, indicating that the ion matched the theoretical *m*/*z* and that S126 was the *O*-glycosylation site (Figure 5D). The *m*/*z* of the *O*-glycopeptide TPILPQ was 1033.5273, while the theoretical *m*/*z* of the unmodified peptide segment was 667.3904. Therefore, the modification was also GalGalNAc (*m*/*z* 365). The corresponding MS/MS spectrum showed the y ions (y5) at *m*/*z* 567.35, which matched the theoretical *m*/*z*, proving that T134 was the *O*-glycosylation site (Figure 5F). Subsequently, we examined peptides containing the core GalNAc (*N*-acetylhexosamine, HexNAc) residue to try and localize sites using fragment ions bearing incomplete glycan chains. Specifically, the glycopeptide SKAPPPSLPSP had a found *m*/*z* of 1442.7240 while the theoretical *m*/*z* of the unmodified peptide segment is 1076.5866, indicating the presence of a core 1 *O*-glycan. Although direct site localization via b-/y-ions was not possible, we observed a b3 + N fragment ion (*m*/*z* 490.26) (Figure 5B). The observed mass increase of 203 Da relative to the unmodified b3 ion (*m*/*z* 287.17) is consistent with the presence of a single HexNAc residue. Therefore, *O*-glycosylation occurs at Ser115. The glycopeptide SLPSP had a found *m*/*z* of 865.4034, while the theoretical *m*/*z* of the unmodified peptide segment is 499.2642, indicating the presence of a core 1 *O*-glycan. Although direct site localization via b-/y-ions was not possible, we observed a b2 + N fragment ion (*m*/*z* 404.20) (Figure 5C). Again, the observed mass increase of 203 Da relative to the unmodified b2 ion (*m*/*z* 201.12) is consistent with the presence of a single HexNAc residue. Therefore, *O*-glycosylation occurs at Ser121. We attempted *O*-glycosylation site localization at Ser114 and Ser132 by analyzing the glycopeptides SSKAPPP (containing two core 1 structures, Figure 5A) and SDTPILPQ (containing one core 1 structure, Figure 5E), respectively. However, we did not achieve precise site assignment using b-ions bearing incomplete glycan chains. Consequently, we based the site assignments to Ser114 and Ser132 solely on the known specificity of the OpeRATOR enzyme. Overall, we directly localized three *O*-glycosylation sites through HCD fragmentation combined with OpeRATOR enzymatic digestion and assigned the remaining two sites based on inference.

One potential benefit of the recently described *O*-glycoprotease OpeRATOR is the reported ability to localize *O*-glycosylation sites using collisional dissociation due to the *N*-terminal location of modified sites within the sequence. Indeed, previous research dismissed the need for electron-driven dissociation for site-specific analysis when using OpeRATOR. So far, *O*-glycosite localization in OpeRATOR-derived *O*-glycopeptides has relied on HCD fragmentation and the assumption that the total glycan mass observed came from a single glycosylated serine or threonine at the *N*-terminus. Besides, HCD preferentially cleaves glycosidic bonds over peptide bonds, creating analytical challenges. Indeed, complete gas-phase deglycosylation may occur; therefore, the absence of glycan-bearing fragments in MS/MS spectra cannot conclusively rule out initial glycosylation. This is further complicated by the characteristic loss of diagnostic glycan-containing b-/y-ions in conventional HCD analysis. Current localization confidence, therefore, primarily depends on two criteria: (1) identification of peptide backbone fragments with a single candidate *O*-glycosylation site and (2) retention of at least one core GalNAc residue of fragment ions as glycosylation evidence. Consequently, the structural characteristics of CTP regions, where adjacent serine/threonine (S/T) residues frequently create multiple potential *O*-glycosylation sites within individual glycopeptides, critically limit our approach. Notably, two of the identified glycopeptides contained multiple clustered S/T residues rather than isolated modification sites, not fulfilling the two criteria. This is despite our MS/MS-based analysis incorporating OpeRATOR’s enzymatic specificity (particularly its *N*-terminal cleavage preference for amino acids with core 1 glycans). These glycopeptides required further investigation. Therefore, even when leveraging enzymatic cleavage patterns for site prediction, resolving ambiguities arising from co-existing or adjacent modification sites requires definitive verification through electron-driven dissociation techniques.

### 2.3. Localizing O-Glycosylation Sites by EThcD

**Figure 6:**
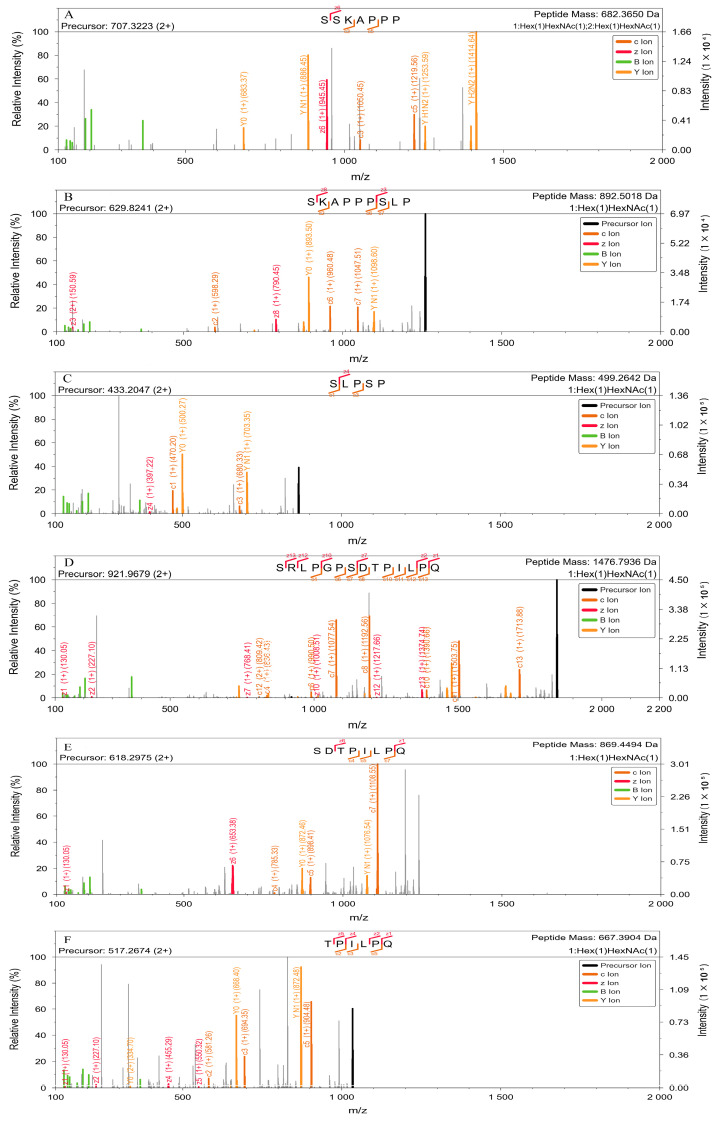
EThcD fragmentation MS/MS spectra of *O*-glycopeptide treated with OpeRATOR and sialidase. EThcD fragmentation MS/MS spectra of the *O*-glycopeptides SSKAPPP (**A**), SKAPPPSLP (**B**), SLPSP (**C**), SRLPGPSDTPILPQ (**D**), SDTPILPQ (**E**), and TPILPQ (**F**).

In 2017, Yu et al. proposed an EThcD fragmentation approach combining data from ETD (favoring peptide fragmentation) and HCD (favoring glycan fragmentation) into one contiguous, more informative spectrum [41]. EThcD has proven particularly valuable for localizing glycosylation sites, primarily owing to its ability to preserve the glycan moieties during fragmentation, thus producing peptidic ions that retain the glycans, displaying characteristic mass shifts. Nicholas et al. pointed out that electron-based dissociation methods are necessary to capture the *O*-glycopeptide diversity produced by OpeRATOR digestion, especially EThcD methods, for localizing *O*-glycosylation sites [42]. Here, after OpeRATOR digestion, we detected 16 glycopeptides containing core 1 (GalGalNAc) modifications by LC–MS/MS (Table 3, Figure 6). We investigated four *O*-glycosylation sites (S115, S121, S126, and T134), also identifiable by HCD fragmentation. The glycopeptides containing these sites were SKAPPPSLP, SLPSP, SRLPGPSDTPILPQ, and TPILPQ. The precursor and peptide mass values indicated the presence of a single *O*-glycan modification on each glycopeptide. Next, we localized the glycosylation sites by MS/MS fragmentation: we identified S115 by the presence of the z8 fragment ion (*m*/*z* 793.45, Figure 6B), S121 by the presence of the z4 fragment ion (*m*/*z* 397.22, Figure 6C), S126 by the z13 fragment ion (*m*/*z* 1374.74, Figure 6D), and T134 by the z5 fragment ion (*m*/*z* 550.32, Figure 6F). Notably, all four identified sites are the *N*-terminal amino acids of their respective peptides, consistent with the specificity of OpeRATOR enzymes. For site S132, which could not be directly confirmed by HCD fragmentation alone, we selected the same glycopeptide SDTPILPQ. Analysis of the MS/MS spectrum revealed a z6 fragment ion (*m*/*z* 653.38, Figure 6E), definitively demonstrating core glycan attachment to the *N*-terminal serine residue. The S114 glycosylation site presented a greater challenge due to its close proximity to S115. To address this, we utilized a missed-cleavage glycopeptide, SSKAPPP, which also corresponds to a peptide selected through HCD. This glycopeptide contained two core 1 structures. The presence of the z6 fragment ion (*m*/*z* 945.45, Figure 6A) enabled the simultaneous and unambiguous localization of glycosylation on both S114 and S115. Other glycopeptides listed in Table 3 exhibited multiple missed cleavage sites. For example, glycopeptide SSKAPPPSLPSP, which contains three miscleavage sites, allowed the identification of glycosylation sites S114, S115, and S121. Furthermore, the identification of the glycopeptide SKAPPPSLP, resulting from enzymatic cleavage between P123 and S124, suggests that S124 is a potential *O*-glycosylation site. The identification of glycopeptides SSKAPPPSLPSP and SPSRLPGPSDTPILPQ confirmed this result. Collectively, these results indicate that S124 represents a novel *O*-glycosylation site within these peptides.

In EThcD, the ETD step is followed by HCD applied to the remaining ions or to enhance the ETD-generated fragments. This sequential approach might not yield as many fragments as HCD alone. The ETD step might consume some precursor ions, leaving fewer ions for HCD to fragment. However, EThcD dissociation occurs at the peptide backbone rather than at glycosidic bonds, preserving labile glycan-peptide bonds and allowing glycopeptide identification glycosylation site assignment. We identified *O*-glycosylation exclusively at *N*-terminal amino acids of the identified glycopeptides, which supports the high specificity of the OpeRATOR enzyme and validates the HCD and OpeRATOR combination for glycosylation site prediction. Furthermore, while HCD fragmentation was insufficient to definitively identify glycosylation at S114 and S132, EThcD fragmentation of the same peptides allowed the unambiguous localization of these *O*-glycosylation sites. Thus, our results indicate that the HCD/OpeRATOR approach is limited for confidently identifying *O*-glycosylation sites on sequences closely resembling CTP-like S/T motifs and warrants further validation using EThcD fragmentation. Furthermore, EThcD fragmentation revealed the macroheterogeneity of *O*-glycosylation, reflecting site-specific heterogeneity and enabling the identification of novel, potentially glycosylated sites.

Removing sialic acid using sialidase and OgpA simplifies the MS/MS spectra, facilitating *O*-GalNAc site localization; however, it also removes information regarding the specific Neu5Ac location on the glycan structures. This limitation is inherent to the approach, as the precise position of sialic acid within the *O*-glycans contributes to the microheterogeneity observed in *O*-glycosylation, which significantly influences the biological function of FSH-CTP. Our primary aim here was to establish a robust and efficient methodology for identifying all *O*-glycosylation sites (*O*-glycan microheterogeneity) on FSH-CTP. Therefore, fully characterizing the sialylation patterns requires further studies. Future work could explore the combination of EThcD fragmentation with trypsin without prior sialidase treatment, analyze trypsin-digested peptides, digest the CTP using the *O*-glycopeptidase IMPa without sialidase treatment, and map the Neu5Ac residues within the *O*-glycan structures. These approaches should provide a more comprehensive understanding of the complete *O*-glycosylation landscape of FSH-CTP, including the critical role of sialylation in its function and bioactivity.

## 3. Materials and Methods

### 3.1. O-Linked Glycan Liberation and Labeling

We labeled *O*-glycans using an EZGlyco *O*-glycan Prep Kit (Tokyo, Japan) [43,44]. Briefly, we mixed 10 µL of FSH-CTP (Elonva biosimilar, produced in CHO cells by Suzhou Shengji Pharmaceutical Co., Ltd., Suzhou, China, Initial concentration: 1.6 mg/mL) solution (10 mg/mL in H_2_O) with 5 µL of glycan-released reagent A and 10 µL of glycan-released reagent B and incubated the mixture at 37 °C for 75 min. Subsequently, we captured the released glycans using glycan-capturing beads, which we washed with acetonitrile. Thereafter, we added 4 mg of 2-aminobenzeamide (2-AB) and 0.04 mg of reducing reagent in 50 µL of methanol/acetic acid/H_2_O (9/2/9) to the beads. We recovered the *O*-glycan-containing solution by centrifugation at 3000× *g* for 1 min. We then incubated the solution at 50 °C for 2.5 h and eluted it with acetonitrile on a cleanup column to remove excess reagent. Next, we diluted the 2-AB-labeled *O*-glycans twice using acetonitrile for LC–MS analysis.

### 3.2. O-GalNAc Glycopeptide Truncation

A 500 µg aliquot of the FSH-CTP sample (Elonva biosimilar, produced in CHO cells by Suzhou Shengji Pharmaceutical Co., Ltd., Suzhou, China; initial concentration: 1.6 mg/mL) was diluted to 1 mg/mL with 8 mol/L urea denaturing buffer. The sample was then heated at 65 °C for 10 min to denature the peptide, cooled to room temperature, and subsequently treated with 30 µL of 1 M DTT (Sigma-Aldrich, Steinheim, Germany) solution at 37 °C for 1 h. Next, 20 µL of 1 M IAM (Sigma-Aldrich, Steinheim, Germany) solution was added, and the mixture was stirred at room temperature in the dark for 30 min before ultracentrifugation at 12,000 rpm for 10–15 min. The ultrafiltration tube was then washed 3–4 times with 300 µL of 20 mM Tris (MP Biomedicals, Santa Ana, CA, USA) buffer (pH 6.8) via ultracentrifugation at 14,000 rpm for 10–15 min per wash. Finally, the sample concentration was measured and adjusted to 1.0 mg/mL with 20 mM Tris buffer (pH 6.8). In the OpeRATOR-truncated scheme, the enriched *O*-glycopeptides were co-treated with OpeRATOR (Genovis, Kävlinge, Sweden) and sialidase at 37 °C overnight.

### 3.3. Glycan Profiling by UPLC-HILIC-FLR-MS and MS/MS

We separated 2-AB-labeled *O*-glycans (1 μL) by UPLC using an ACQUITY UPLC glycan BEH amide column (2.1 mm × 150 mm, 1.7 μm) from Waters on an I-Class ACQUITY instrument (Waters, Milford, MA, USA). Solvent A was a 50 mM ammonium formate buffer (pH 4.4), and solvent B was acetonitrile. The eluent gradient was: 0–5 min, 85% solvent B; 5–20 min, 85–50% solvent B; 20–25 min, 50% solvent B; 25.1–35 min, 85% solvent B. The flow rate was 0.25 mL/min, and the column temperature was 45 °C. The fluorescence detector excitation and emission wavelengths were 320 and 420 nm, respectively. The fluorescence detector was coupled with a Waters UPLC I-Class/synapt G2-S QTOF mass spectrometer (Waters). We acquired full-scan mass spectra in positive ion mode with a scan range of *m*/*z* 50 to 2000. For MS measurements, the electrospray voltage was 3.5 kV, and the heat capillary temperature was 250 °C. We analyzed the MS and fluorescence chromatograms using UNIFI (Waters). We performed MS/MS on a Waters UPLC I-Class system coupled to a Synapt G2-S QTOF (Waters)mass spectrometer equipped with an ESI source in positive ion mode. We used collision-induced dissociation for fragmentation in the MS/MS experiments. We fragmented precursor ions at *m*/*z* 504.22 (retention time 0–10 min) with a collision energy of 5–25 V and scanned product ions at *m*/*z* 50–2000. We fragmented precursor ions at *m*/*z* 795.32 (retention time 10–14.5 min) with a collision energy of 10–30 V and scanned product ions at *m*/*z* 50–2000. We fragmented precursor ions at *m*/*z* 1086.41 (retention time 14.5–20 min) with a collision energy of 20–40 V, with product ions scanned at *m*/*z* 50–2000.

### 3.4. LC-MS/MS Analysis with HCD

We performed LC-MS/MS analysis with HCD for *O*-GalNAc glycopeptides on a Vanquish Flex LC system coupled online to an Orbitrap Exploris 480 (Thermo Fisher Scientific, Waltham, MA, USA). We separated glycopeptidic samples (2 μL) by UPLC using an ACQUITY UPLC BEH C18 column (2.1 mm × 150 mm, 1.7 μm, 130 Å). The flow rate was 0.2 mL/min with buffer A (water, 0.1% formic acid) and buffer B (acetonitrile, 0.1% formic acid). The elution gradient was: 0–3 min, 1% solvent B; 3–10 min, 1–7% solvent B; 10–80 min, 7–32% solvent B; 80–85 min, 32–90% solvent B; 85–90 min, 90% B; 90.1–100 min, 1% B. The column temperature was 60 °C. We acquired MS/MS spectra (HCD fragmentation) in positive ion mode with a scan range of *m*/*z* 300 to 2000. The first-level resolution was 60,000, and the second-level resolution was 15,000. The electrospray voltage and heat capillary temperature were 3.8 kV and 320 °C. AGC level 1 was set to 300% and level 2 to 100%. The collision energy was 30%.

### 3.5. LC-MS/MS with EThcD

We performed LC-MS/MS with EThcD for *O*-GalNAc glycopeptides in a Vanquish Flex LC system coupled online to an Orbitrap Eclipse™ Tribrid™ (Thermo Fisher Scientific, USA). We prepared glycopeptide samples (2 μL) by UPLC using an ACQUITY UPLC BEH C18 column (2.1 mm × 150 mm, 1.7 μm, 130 Å). The flow rate was 0.2 mL/min with buffer A (water, 0.1% formic acid) and buffer B (acetonitrile, 0.1% formic acid). The eluent gradient was: 0–9 min, 3% solvent B; 9–90 min, 32–90% solvent B; 90–90.1 min, 90–3% solvent B; 90.1–100 min, 3% solvent B. The column temperature was 60 °C. We acquired MS/MS spectra (HCD fragmentation) in positive ion mode with a scan range of *m*/*z* 300 to 2000. The first-level resolution was 60,000, and the second-level resolution was 15,000. The electrospray voltage and heat capillary temperature were 3.6 kV and 320 °C, respectively. AGC level 1 was set to 300% and level 2 to 100%. The collision energy was at 30%, the RF Lens was 40, ETD SA% was 27, and EThcD AGC was 800%.

### 3.6. Data Analysis

We compared glycopeptide MS/MS fragment spectra with selected protein and glycan databases using Pglyco 3.1 software [45] (https://github.com/pFindStudio/pGlyco3/releases, accessed on 7 April 2024). We set precursor and fragment mass tolerance to 10 and 20 ppm, respectively. As the protein from the databases were digested with the OpeRATOR enzyme, the specific or semi-specific cleavage occurred at the *N*-terminus of Ser/Thr, with up to three missed cleavages. We set methionine oxidation (M, +15.995 Da) and asparagine/glutamine deamidation (N/Q, +0.9804 Da) as variable modifications. We set GalGalNAc (HexNAc(1)Hex(1)), NeuAcGalGalNAc (HexNAc(1)Hex(1)NeuAc(1)), and NeuAcGal(NeuAcGalNAc) (HexNAc(1)Hex(1)NeuAc(2)) as validations for the identified glycopeptide modifications. We filtered the searches using strict criteria (1% FDR). We processed and visualized the software-analyzed data using GP-Plotter 1.0.1. We performed statistical analysis and produced graphics with GraphPad Prism 10.1.2.

## 4. Conclusions

This study presents an analytical strategy for characterizing the *O*-linked glycan profile and glycosylation sites of rhFSH-CTP. *O*-glycan profiling utilizing established methods and the commercially available EZGlyco *O*-glycan Prep Kit for glycan release and 2-AB labeling demonstrated reproducible and stable results. It revealed that the predominant *O*-glycoforms were core 1-based structures; however, we did not evaluate the sensitivity nor compare it with alternative *O*-glycan release techniques.

For *O*-glycosylation site localization, we employed the *O*-glycopeptidase OpeRATOR, which selectively cleaves at the *N*-termini of *O*-glycosylated Ser or Thr residues. Optimal OpeRATOR performance required prior removal of terminal NeuAc units using sialidase. Because of this requirement and the *O*-glycan profiling results, we focused on identifying glycopeptides bearing core 1 *O*-glycans. While HCD fragmentation, combined with OpeRATOR digestion, successfully localized some glycosylation sites, the adjacent serine/threonine residues within the CTP sequence limited the analysis. This resulted in ambiguities in site assignment for certain peptides that we ultimately clarified through EThcD fragmentation. EThcD analysis, coupled with OpeRATOR digestion, allowed us to identify all six glycosylation sites unambiguously. However, although OpeRATOR and sialidase treatment improved spectra interpretation, they necessarily restricted our ability to fully assess site-specific heterogeneity and the extent of *O*-glycan site occupancy.

This approach offers an analysis strategy for *O*-linked glycan profiling and *O*-glycosylation site identification in FSH-CTP biosimilars and related CTP-fusion proteins. Thus, it provides a valuable reference for improving drug quality standards and addressing the current lack of standardized *O*-glycan identification methods. Further optimization of complete characterization of site-specific heterogeneity and occupancy, however, could enhance the comprehensiveness of the analysis.

## Figures and Tables

**Table 1:** Glycan names and structures.

Time (min)	Glycan Name	Glycan Structure ^1^	*m*/*z*
7.98	GalGalNAc	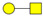	504.22
12.94	NeuAcGalGalNAc	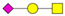	795.32
13.77	Gal(NeuAcGalNAc)	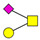	795.32
15.97	NeuAcGal(NeuAcGalNAc)	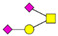	1086.41

^1^ The illustrated glycan structures omit the 2-AB fluorophore.

**Table 2:** Accuracy evaluation results of the method (recovery rate %).

Glycan Name	10 μg (%)	20 μg (%)	60 μg (%)	100 μg (%)
GalGalNAc	123	114	87	80
NeuAcGalGalNAc	127	118	81	75
Gal(NeuAcGalNAc)	128	113	82	75
NeuAcGal(NeuAcGalNAc)	123	109	96	94
Total glycans	126	115	86	80

**Table 3:** FSH-CTP glycopeptides with *O*-GalNAc: EThcD MS/MS after OpeRATOR/sialidase treatment.

Peptide Sequence ^1^	Location	Glycosylation Sites ^2^	Precursor (*m*/*z*; Charge)	Peptide Mass (Da)	Positioned Fragment Ions (*m*/*z*)
SKAPPPSLP	115–123	S115	629.8241 (2+)	893.5018	z8 793.45
SLPSP	121–127	S121	433.2047 (2+)	499.2642	z4 397.22
SRLPGPSDTPILPQ	126–139	S126	921.9679 (2+)	1476.7936	z13 1374.74
SDTPILPQ	132–139	S132	618.2975 (2+)	869.4464	z6 653.38
TPILPQ	134–139	T134	517.2674 (2+)	677.3904	z5 550.32
SLPSPSRLPGP	121–131	S121	736.8767 (2+)	1106.6084	z10 1004.56
SSKAPPP	114–120	S114, S115	707.3223 (2+)	682.3650	z6 945.45
SSKAPPPSLP	114–123	S114, S115	855.9053 (2+)	979.5338	z9 1242.62
SSKAPPPSLP	114–123	S114, S121	855.9045 (2+)	979.5338	z8 1155.59; z9 1243.62; c7 1047.52; c8 1499.68
SKAPPPSLPSP	114–125	S115, S121	645.9761 (2+)	1076.5866	c2 300.15 (2+); c6 960.48; c7 1412.65
SRLPGPSDTPILPQ	126–139	S126, S132	1104.5358 (2+)	1476.7936	c4 836.43; c6 990.50; c7 1442.66
SLPSPSRLPGPSDTPILPQ	121–139	S121, S132	897.1101 (3+)	1958.0472	c1 470.20; c11 1472.76; c15 1119.04 (2+)
SSKAPPPSLPSP	114–125	S114, **S124**	947.9465 (2+)	1163.6186	z10 1341.68; z11 1428.71; c7 1048.52
SSKAPPPSLPSP	114–125	S114, S115, S121	754.0142 (3+)	1163.6186	c1 470.20; c2 922.36; c7 1413.65; c8 1865.81
SRLPGPSDTPILPQ	126–139	S126, S132, T134	858.4017 (3+)	1476.7936	c1 470.20; c7 1443.66; z6 1016.51
SPSRLPGPSDTPILPQ	124–139	**S124**, S126, S132	919.7639 (3+)	1660.8784	c2 567.25; c3 1019.41; z7 766.40; z9 1315.62

^1^ The *O*-glycosylation sites are underlined (modified glycan: core 1). ^2^ The novel *O*-glycosylation sites (S124) are in bold.

## Data Availability

The raw data supporting the conclusions of this article will be made available by the authors upon request.

## Supplementary Materials

The following supporting information can be downloaded at: https://www.mdpi.com/article/10.3390/molecules30102141/s1, Figure S1. Mass spectra of the four main glycan types; Figure S2. Specificity (blank solution) chromatogram; Figure S3. Chromatogram with the flow rate adjusted to 0.1 mL/min; Table S1. Peak area percentages (%) for the four glycoforms, normalized to the total peak area, at different initial protein amounts; Table S2. Peak area percentages (%) for the four glycoforms and byproducts, normalized to the total peak area, at different initial protein amounts; Figure S4. The MS/MS spectrum of SLPSPSRLPGP acquired with EThcD fragmentation; Figure S5. The MS/MS spectrum of SSKAPPPSLP acquired with EThcD fragmentation; Figure S6. The MS/MS spectrum of SSKAPPPSLP acquired with EThcD fragmentation; Figure S7. The MS/MS spectrum of SKAPPPSLPSP acquired with EThcD fragmentation; Figure S8. The MS/MS spectrum of SRLPGPSDTPILPQ acquired with EThcD fragmentation; Figure S9. The MS/MS spectrum of SLPSPSRLPGPSDTPILPQ acquired with EThcD fragmentation; Figure S10. The MS/MS spectrum of SSKAPPPSLPSP acquired with EThcD fragmentation; Figure S11. The MS/MS spectrum of SSKAPPPSLPSP acquired with EThcD fragmentation; Figure S12. The MS/MS spectrum of SRLPGPSDTPILPQ acquired with EThcD fragmentation; Figure S13. The MS/MS spectrum of SPSRLPGPSDTPILPQ acquired with EThcD fragmentation.
