# Supplementary material for: Analysis Strategy for Identifying the O-Linked Glycan Profile and O-glycosylation Sites on Recombinant Human Follicle Stimulating Hormone-C-terminal Peptide (rhFSH-CTP)
Source: Molecules. 2025 May 13;30(10):2141. doi: 10.3390/molecules30102141 (PMC12114138; doi:10.3390/molecules30102141)
Supplement: Supplementary file 1 [file molecules-30-02141-s001.zip › molecules-3612074-supplementary.pdf]

SUPPORTING INFORMATION

Analysis Strategy for Identifying the *O*-Linked Glycan Profile and *O*-glycosylation Sites on Recombinant Human Follicle Stimulating Hormone-*C*-terminal Peptide (rhFSH-CTP)

Xinyue Hu[a†], Yuxing Xiang[b†], Xiaoming Zhang[a], Yue Sun[a], Yi Li[a], Lvyin Wang[a], Ping Lv [a], Zhen Long[c], Chenggang Liang[a], Jing Li[a*]

a. National Institutes for Food and Drug Control, Beijing, 102629, China

b. China Pharmaceutical University, Nanjing, 210009, China

c. ThermoFisher scientific corporation, Beijing, 100080, China

* Corresponding author: National Institutes for Food and Drug Control, China. E-mail address: li_jing@nifdc.org.cn (Jing Li)

[†]These authors made equal contribution to this work.

(1) Figure S1. Mass spectra of the four main glycan types.
(2) Figure S2. Specificity (blank solution) chromatogram
(3) Figure S3. Chromatogram with the flow rate adjusted to 0.1 mL/min
(4) Table S1 Peak area percentages (%) for the four glycoforms, normalized to the total peak area, at different initial protein amounts
(5) Table S2 Peak area percentages (%) for the four glycoforms and byproducts, normalized to the total peak area, at different initial protein amounts
(6) Figure S4 The MS/MS spectrum of SLPSPSRLPGP acquired with EThcD fragmentation
(7) Figure S5 The MS/MS spectrum of SSKAPPPSLP acquired with EThcD fragmentation
(8) Figure S6 The MS/MS spectrum of SSKAPPPSLP acquired with EThcD fragmentation
(9) Figure S7 The MS/MS spectrum of SKAPPPSLPSP acquired with EThcD fragmentation
(10) Figure S8 The MS/MS spectrum of SRLPGPSDTPILPQ acquired with EThcD fragmentation
(11) Figure S9 The MS/MS spectrum of SLPSPSRLPGPSDTPILPQ acquired with EThcD fragmentation
(12) Figure S10 The MS/MS spectrum of SSKAPPPSLPSP acquired with EThcD fragmentation
(13) Figure S11 The MS/MS spectrum of SSKAPPPSLPSP acquired with EThcD fragmentation
(14) Figure S12 The MS/MS spectrum of SRLPGPSDTPILPQ acquired with EThcD fragmentation
(15) Figure S13 The MS/MS spectrum of SPSRLPGPSDTPILPQ acquired with EThcD fragmentation

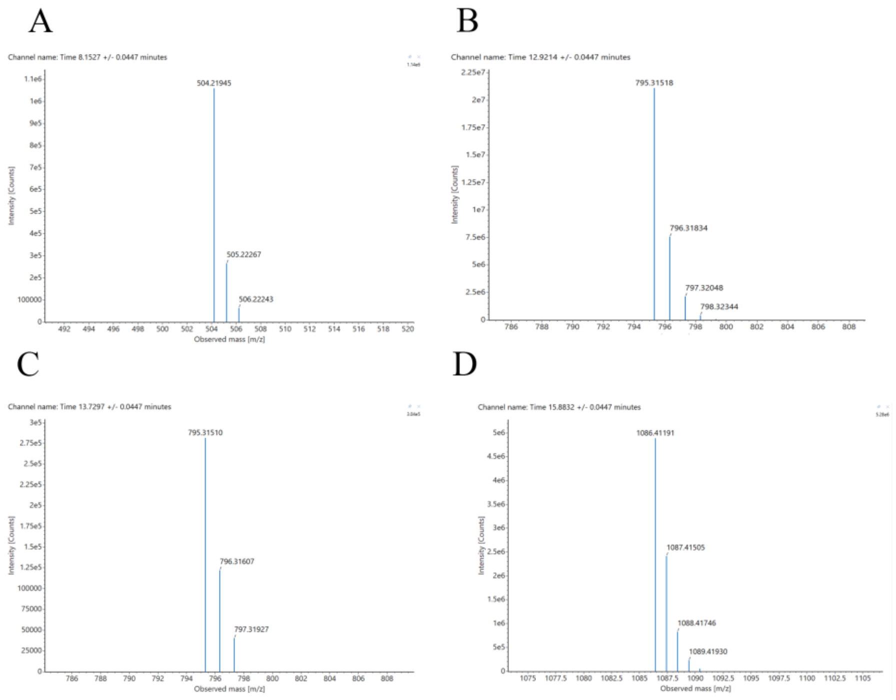

Figure S1. Mass Spectra of the Four Main Glycan Types. A. Mass Spectra of GalGalNAc. B. Mass Spectra of NeuAcGalGalNAc. C. Mass Spectra of Gal(NeuAcGalNAc). D. Mass Spectra of NeuAcGal(NeuAcGalNAc)

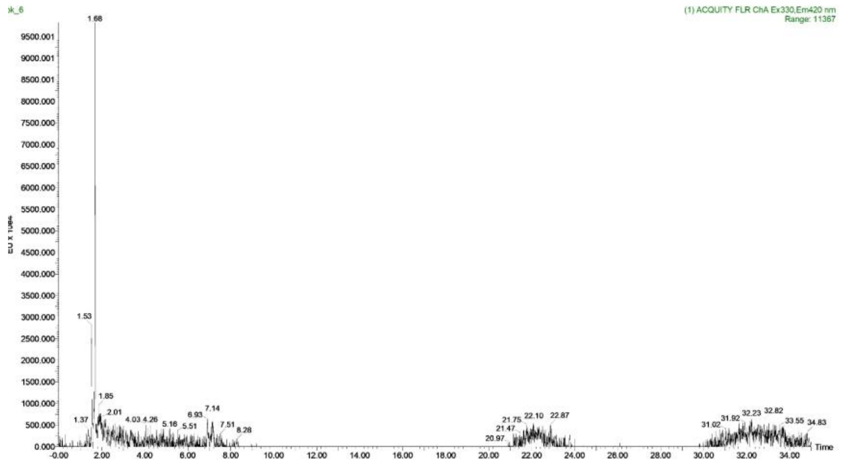

Figure S2 Specificity (blank solution) chromatogram

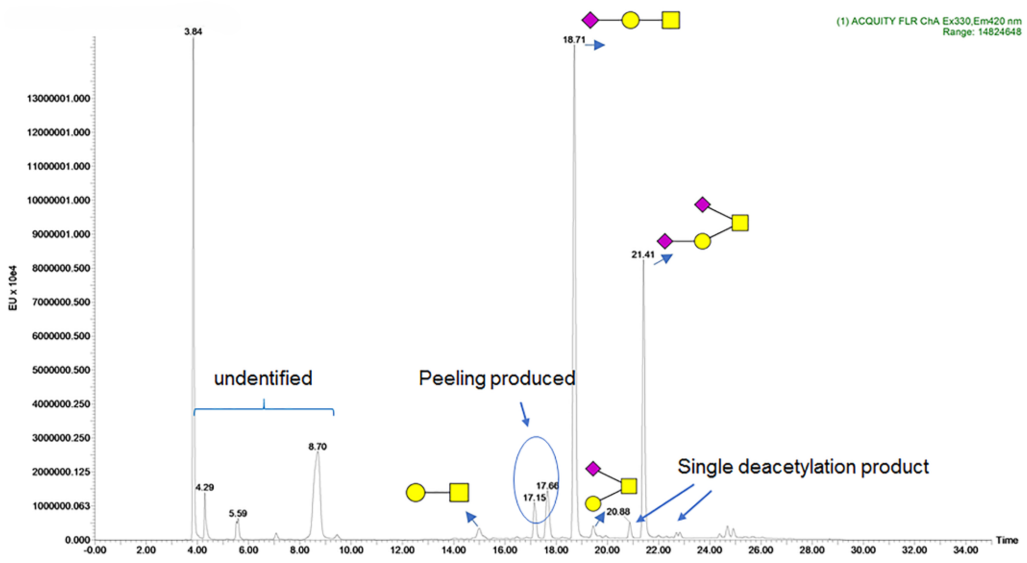

Figure S3 Chromatogram with the flow rate adjusted to 0.1 mL/min

Table S1 Peak area percentages (%) for the four glycoforms, normalized to the total peak area, at different initial protein amounts

initial protein amount	GalNAc Gal	Neu5AcGalGal NAc	Neu5Ac[Gal]Gal NAc	Neu5Ac[Neu5AcGal]Gal NAc
10 µg	1.6	68.0	2.05	28.4
20 µg	1.6	68.8	1.99	27.5
40 µg	1.7	67.2	2.02	29.1
60 µg	1.7	63.7	1.94	32.7
100 µg	1.6	62.5	1.88	34.0

Table S2 Peak area percentages (%) for the four glycoforms and byproducts, normalized to the total peak area, at different initial protein amounts

initial protein amount	GalNAcGal	peeling	Neu5AcGal GalNAc	Neu5Ac[Gal] GalNAc	single deacetylation 1	Neu5Ac[Neu5Ac Gal]GalNAc	single deacetylation 2
10 µg	1.3	10.9	58.5	1.6	2.0	24.2	1.4
20 µg	1.3	10.9	59.5	1.6	1.9	23.6	1.3

40 µg	1.3	10.9	58.3	1.6	1.7	25.0	1.3
60 µg	1.2	11.1	55.2	1.7	1.6	28.1	1.2
100 µg	1.2	11.2	54.0	1.7	1.5	29.3	1.1

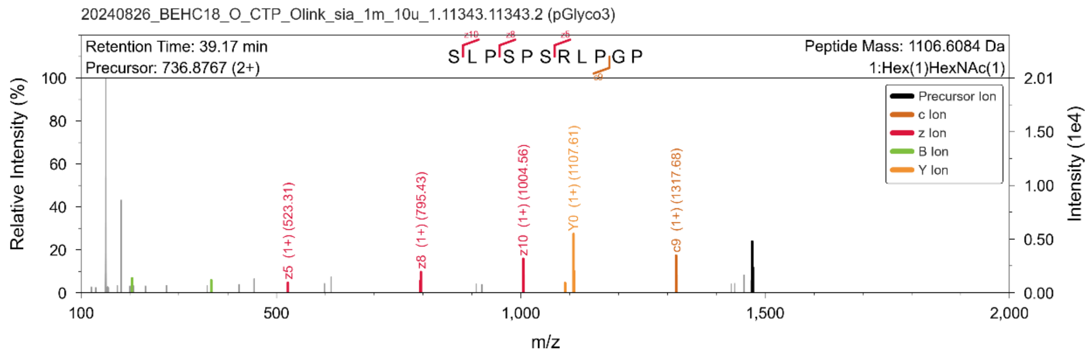

Figure S4 The MS/MS spectrum of SLPSPSRLPGP acquired with EThcD fragmentation

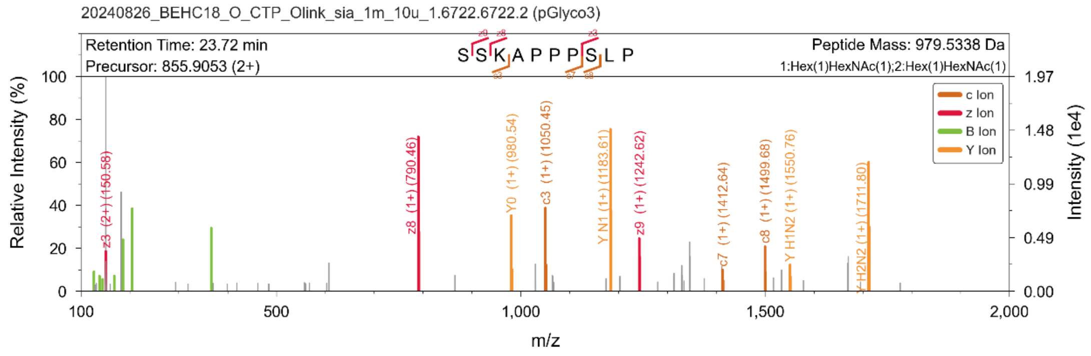

Figure S5 The MS/MS spectrum of SSKAPPPSLP acquired with EThcD fragmentation

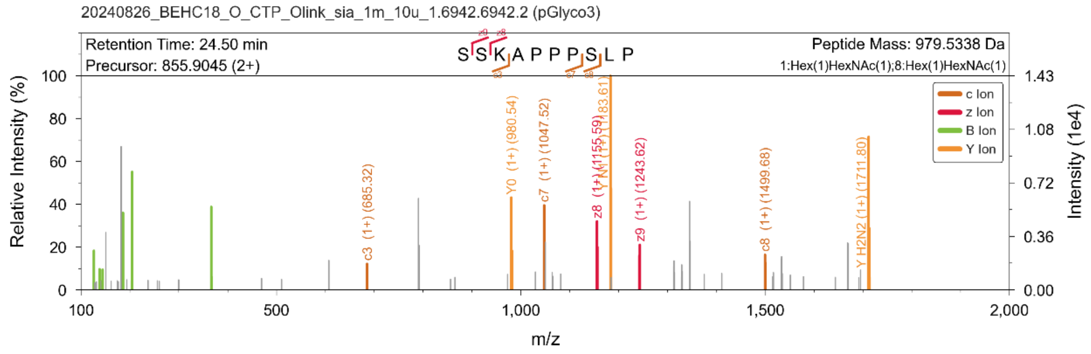

Figure S6 The MS/MS spectrum of SSKAPPPSLP acquired with EThcD fragmentation

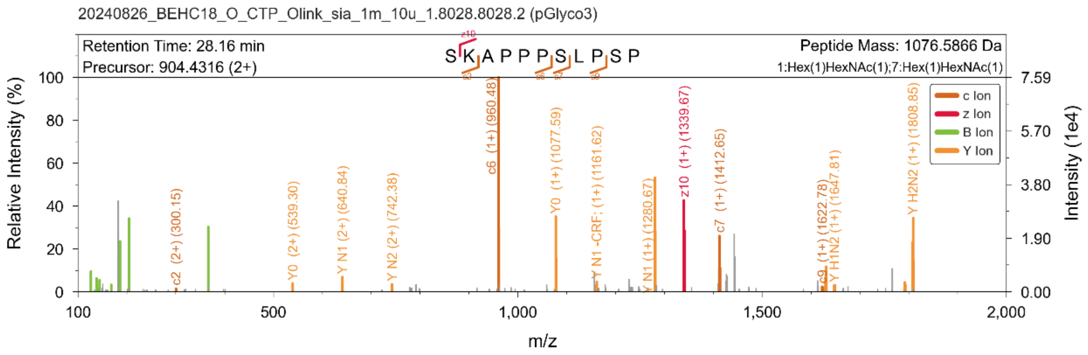

Figure S7 The MS/MS spectrum of SKAPPPSLPSP acquired with EThcD fragmentation

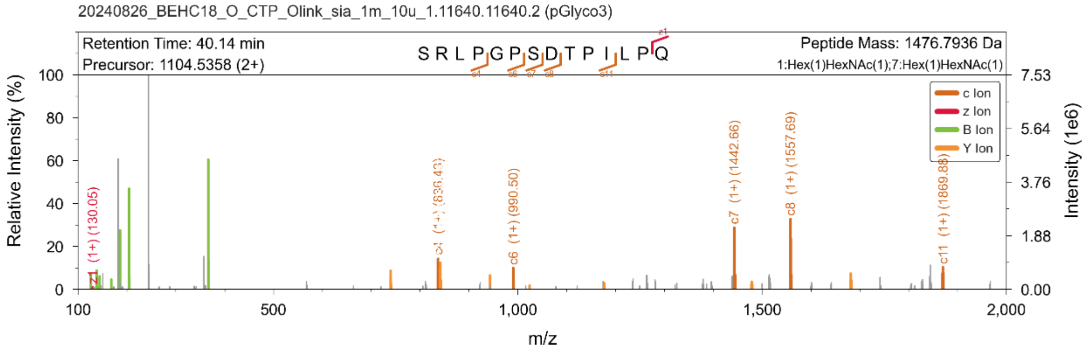

Figure S8 The MS/MS spectrum of SRLPGPSDTPILPQ acquired with EThcD fragmentation

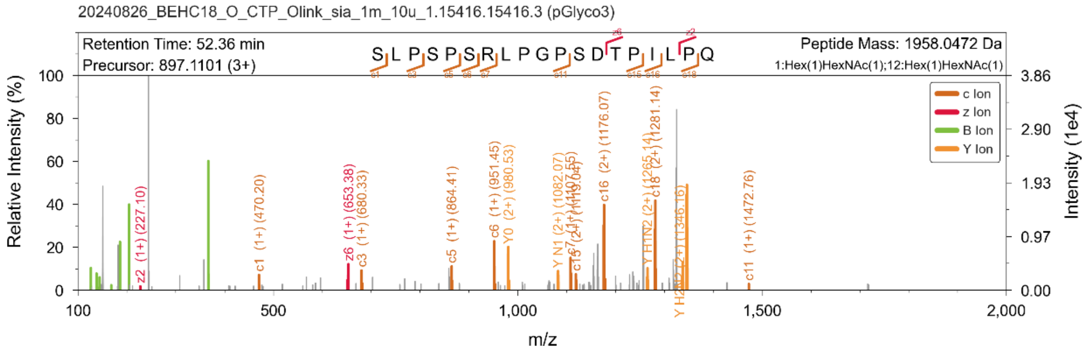

Figure S9 The MS/MS spectrum of SLPSPSRLPGPSDTPILPQ acquired with EThcD fragmentation

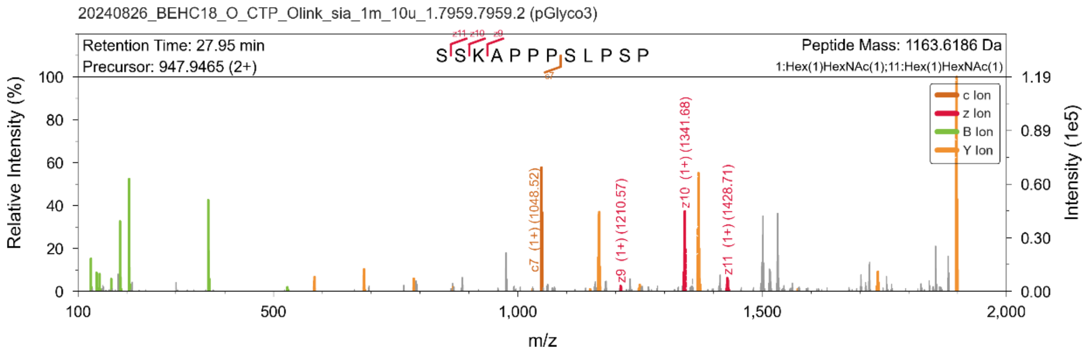

Figure S10 The MS/MS spectrum of SSKAPPPSLPSP acquired with EThcD fragmentation

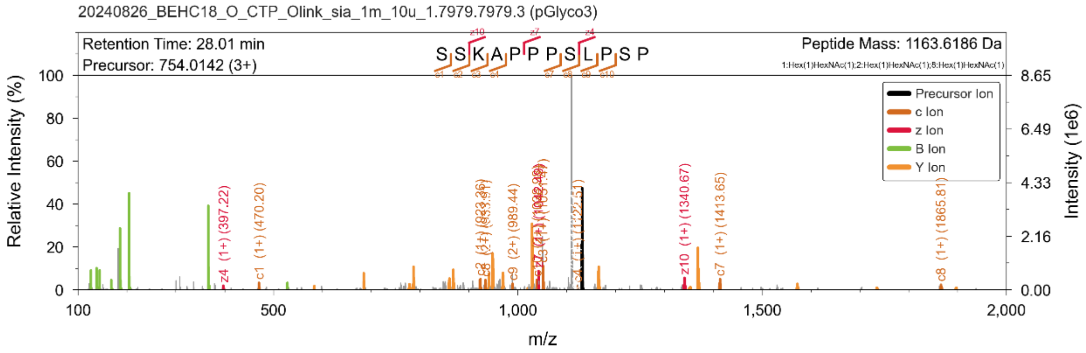

Figure S11 The MS/MS spectrum of SSKAPPPSLPSP acquired with EThcD fragmentation

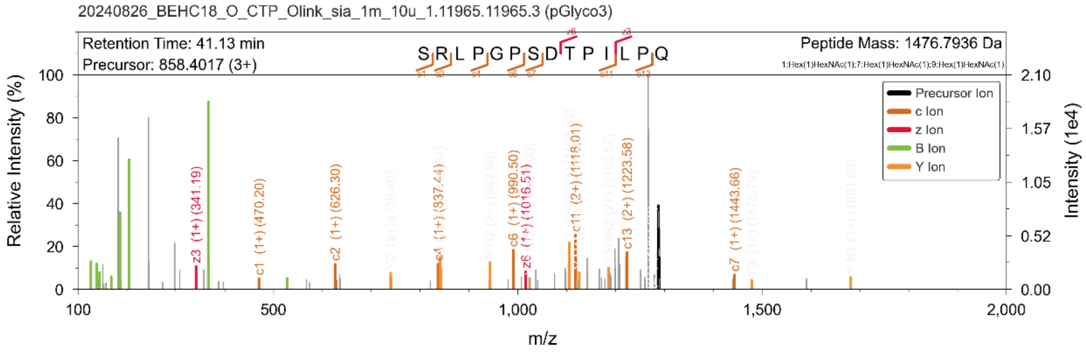

Figure S12 The MS/MS spectrum of SRLPGPSDTPILPQ acquired with EThcD fragmentation

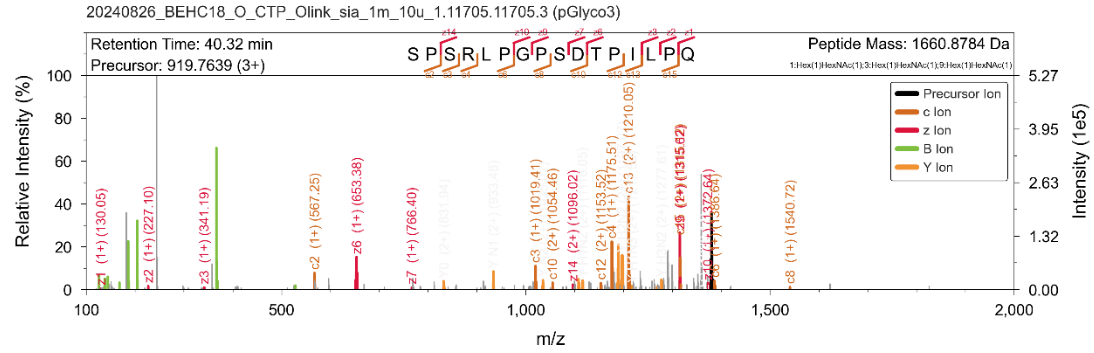

Figure S13 The MS/MS spectrum of SPSRLPGPSDTPILPQ acquired with EThcD fragmentation
